# Intrinsic nutrients and defensive compounds drive coarse woody debris decay in five dominant subtropical tree species, China

**DOI:** 10.1016/j.isci.2025.112961

**Published:** 2025-06-19

**Authors:** Xiaoyu Wang, Tingsi Xie, Hui Chen, Shangbin Bai, Nan Wang

**Affiliations:** 1 Department of Biological Environment, Jiyang College of Zhejiang A&F University, Zhuji, Zhejiang 311800, P.R. China; 2 School of Forestry & Biotechnology, Zhejiang A & F University, Hangzhou, Zhejiang 311300, P.R. China

**Keywords:** soil science, plant biochemistry, soil chemistry, soil ecology, soil biology

## Abstract

Coarse woody debris (CWD) is crucial for carbon and nutrient cycling in forest ecosystems, with decomposition rates influenced by species-specific wood traits. This five-year study in a subtropical Chinese forest evaluated the CWD decomposition of five dominant species. Results showed that broadleaved species decomposed fastest (k = 0.230 years^−1^), followed by moso bamboo (0.168 years^−1^), and conifers slowest (0.022 years^−1^). Fast-decaying species were characterized by high hemicellulose, cellulose, and phosphorus (P) content, with rapid P release within the first decay year and nitrogen (N) accumulation over time. Tannin initially inhibited decay in fast-decaying species, but defensive compounds (tannins and phenolics) showed no sustained effect on decomposition rates. These findings indicate active CWD decomposition in subtropical forests, where P limitation outweighs N for broadleaves and bamboo. Defensive compounds only weakly influenced early-stage decay. Our results enhance the understanding of deadwood decomposition and carbon sink dynamics in subtropical ecosystems.

## Highlights

• Studied the differences in wood decomposition rates among dominant subtropical forest species
• Nitrogen and phosphorus regulate coniferous species decay during early decomposition stages
• Phosphorus is rapidly released in the first year while nitrogen accumulates over time
• Defensive tannins and phenolics have a weak effect to inhibit decay at early stage

## Graphical abstract

**Figure:**
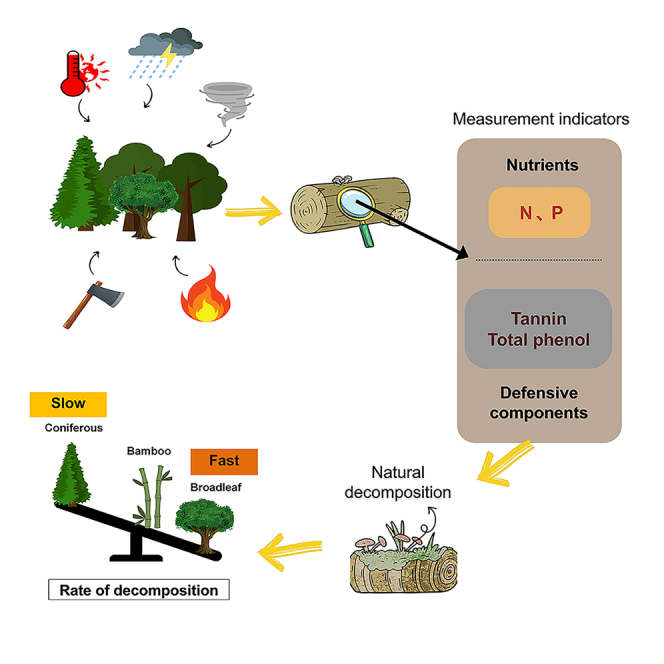

## Introduction

Forests play a critical role in carbon sequestration and nutrient cycling in terrestrial ecosystems.^1^ Woody debris in forests includes snags (standing dead trees), logs, stumps, large branches, and twigs. Following Long-Term Ecological Research (LTER) standards, it is classified as coarse woody debris (CWD, diameter ≥10 cm) and fine woody debris (FWD, diameter >2 cm and <10 cm).^2^ Notably, CWD is a key ecological unit in forests due to its high structural and functional significance.^3^^,^^4^^,^^5^ Through decomposition, CWD mediates carbon transfer from vegetation to soil pools, regulating carbon cycling and balance in forest ecosystems.^6^^,^^7^ Globally, forest CWD stores 20–160 Pg C,^8^ comprising ∼8% of the total carbon pool.^9^^,^^10^ Decay classes serve as a common, time-efficient tool to estimate decay stages, reflect physicochemical changes in CWD, and are fundamental for accurate CWD carbon stock assessment, dynamic nutrient-cycling modeling, and understanding biological community succession.

CWD serves as a critical biodiversity reservoir in forest ecosystems, supporting an estimated 400,000–1,000,000 taxa across its surface and internal matrices. These biological communities include epiphytic flora (e.g., mosses, lichens, and ferns), microbial consortia (bacteria, fungi, archaea), invertebrates, plant seedlings, and vertebrates. As decomposition progresses, these assemblages undergo predictable successional shifts in composition and structure.^11^^,^^12^ For instance, bacterial species richness and diversity increase progressively with decay stage.^13^ CWD carbon stocks also vary significantly among decay classes,^14^ approximating a normal distribution across decomposition stages but exhibiting pronounced spatial heterogeneity.^15^^,^^16^ In most forest ecosystems, CWD in intermediate decay stages (II–III) constitutes a major carbon storage fraction.^17^ By contrast, Class V CWD is largely reduced to a fine, soil-integrated powder with low bulk density and carbon content, resulting in minimal carbon storage.

In forests, CWD is naturally input by tree competition and disturbance-induced forest decline and tree mortality, e.g., wind, snow, fire, insects, debris flows, fungal invasion, and human disturbances such as logging.^18^ Tree mortality is expected to increase and contribute to a larger CWD stock in global forests, as extreme events are predicted to occur with greater frequency and severity in the future.^19^^,^^20^ Therefore, researchers are increasingly concerned about changes in CWD stocks and ecological processes in forest ecosystems under climate change, in addition to their ecological roles.^21^^,^^22^

CWD decomposition is a complex ecological process influenced by intrinsic factors (e.g., substrate quality, including wood chemical and physiological properties), and external factors (e.g., climatic conditions such as temperature/moisture and decomposers such as fungi, bacteria, termites, beetles, and other invertebrates).^23^^,^^24^ Among these, substrate quality plays a critical role in the early decay stages.^25^^,^^26^ Consequently, decomposition rates vary significantly among tree species: higher lignin concentrations and dry-matter content enhance decay resistance. For instance, in the U.S. Pacific Northwest, *Alnus* logs exhibit a decomposition rate (k = 0.08 years^−1^) nearly an order of magnitude higher than *Thuja* logs (k = 0.007 years^−1^).^4^ Generally, angiosperms decompose faster than gymnosperms.

Gymnosperm wood is rich in lignin, which forms a barrier around the lignocellulosic complex due to its complex structure and high concentration. Additionally, gymnosperm logs contain abundant extractable organic compounds (e.g., terpenes and derivatives), which suppress fungal growth and degradation activities.^27^^,^^28^ These factors collectively hinder gymnosperm decomposition.^29^ In contrast, angiosperms are nutrient-dense (e.g., N and P), with high N/P concentration attracting decomposers and accelerating decay.^26^ Intrinsic defensive compounds (e.g., tannins and phenols) also influence decay rate. Cheng et al. showed that tannins alone explained 33–40% of decay rate variation in smooth cord-grass (*Spartina alterniflora Loisel*.).^30^ Tannins and phenolics inhibit decomposition by chelating proteins, toxic effects on microbes, and enzyme activity suppression.^31^^,^^32^ While previous research focused on defensive compounds in litter decomposition, their interactions with nutrient traits in deadwood decomposition remain underexplore.^33^

To answer these questions, we established a forest experimental platform in the subtropical region of southeastern China. The study included two coniferous species: Masson pine (*Pinus massoniana* Lamb.) and Chinese fir (*Cunninghamia lanceolata* (Lamb.) Hook.); two broadleaved species: Cyclobalanopsis glauca (*Quercus glauca* Thunb.) and Schima (*Schima superba* Gardner & Champ.); and moso bamboo (*Phyllostachys edulis*). Although *Phyllostachys edulis* forms extensive bamboo forests in the region, its deadwood decomposition dynamics are poorly understood. Compared to conifers and broadleaves, it exhibits distinct decay patterns due to large internodal cavities, high cortical silica content, and unique lignocellulose composition.^34^

In this study, we performed a five-year *in situ* field experiment on deadwood of five species to analyze dynamic changes in N, P, tannin, and total phenolics during decomposition. We explored the relationships between nutrients/defensive compounds and decay rates across different decay stages. We hypothesized that: (i) decomposition rates would differ significantly among species, with moso bamboo exhibiting an intermediate decay rate between fast-decaying broadleaves and slow-decaying conifers; (ii) in this subtropical ecosystem, high N and P contents in CWD would promote decomposition, whereas elevated tannin and phenolic levels would inhibit it.

## Results

### Decay rate difference among species

**Figure 1:**
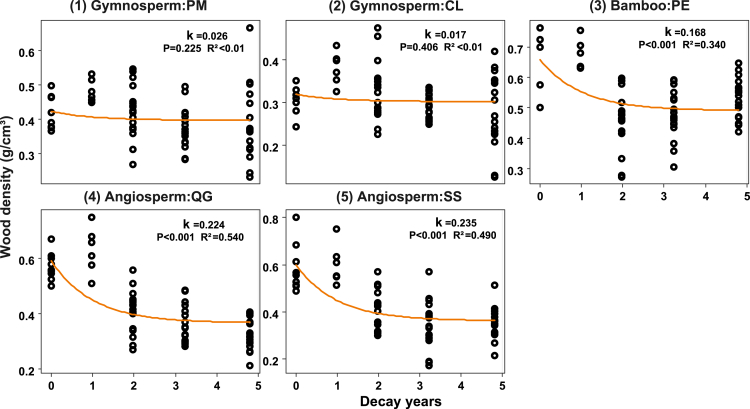
CWD decomposition rates of the five studied species over 5-year period Decomposition rate constant (k) was estimated using an exponential model. *Pinus massoniana* Lamb. (PM); *Cunninghamia lanceolata* (Lamb.) Hook. (CL); *Quercus glauca* Thunb. (QG); *Schima superba* Gardner & Champ. (SS); and *Phyllostachys edulis* (PE).

Over the 5-year decomposition period, the decomposition rate k was *Schima superba* (0.235 years^−1^) > *Quercus glauca* (0.224 years^−1^) > *Phyllostachys edulis* (0.168 years^−1^) > *Pinus massoniana* (0.026 years^−1^) > *Cunninghamia lanceolata* (0.017 years^−1^) (Figure 1). The angiosperm and bamboo deadwood exhibited rapid decomposition in the first two years, with mass loss reaching 53.32%(SS), 46.46% (QG), and 37.84% (PE), respectively.

### N and P change dynamics

**Figure 2:**
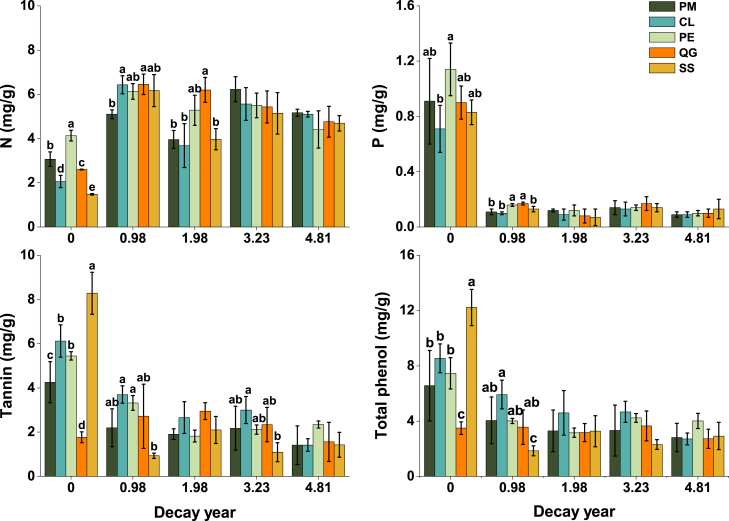
The N, P, tannins, and total phenols content (mean ± SD) in different tree species (i.e., CWD types) along with decay progress Different lower letters indicate statistically significant differences (*p* < 0.05) in the indicators among different tree species at certain decay year.

**Figure 3:**
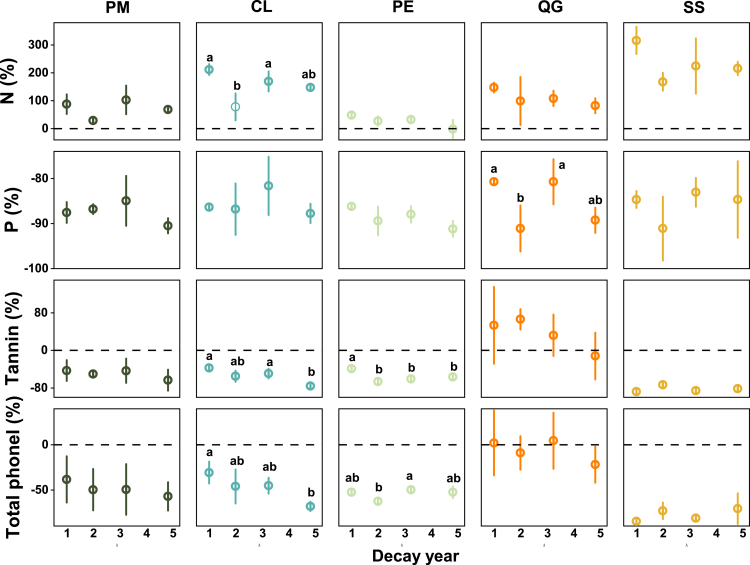
Relative changes (compared to the first sampling) in N, P, tannins, and total phenols with decay process in different tree species (i.e., CWD types) Different lower letters indicate statistically significant differences (*p* < 0.05) in the indicators among different decay times.

N and P contents in CWD were primarily modulated by decomposition time rather than tree species (Tables 1 and 2). After one year of decay, all species showed N accumulated in deadwood, with *Schima superba* exhibiting the highest increase (2.5-fold of initial content) and *Phyllostachys edulis* the lowest (0.49-fold) (Figures 2 and 3). Conversely, P content decreased rapidly within the first year and stabilized thereafter, with no significant interspecific differences (Figures 2 and 3).

**Table 1:** Two-way ANOVA analysis of nutrients (N, P) and defensive compounds (tannin and total phenol) affected by decay year (Y) and tree species (i.e., CWD type, S)

Components	Factors	Df	*F*	*p value*	*PEta.sq*^a^
Nitrogen (N)	Decay year (Y)	4	59.30	<0.001∗∗∗	0.80
	Species (S)	4	2.72	<0.05∗	0.15
	Y×S	16	2.40	<0.001∗∗∗	0.39
Phosphorus (P)	Decay year (Y)	4	208.48	0.008∗∗	0.93
	Species (S)	4	1.93	0.117	0.11
	Y×S	16	1.59	0.100	0.30
Tannin	Decay year (Y)	4	50.35	<0.001∗∗∗	0.77
	Species (S)	4	4.71	0.002∗∗	0.24
	Y×S	16	9.00	<0.001∗∗∗	0.71
Total phenol	Decay year (Y)	4	31.13	<0.001∗∗∗	0.68
	Species (S)	4	3.54	<0.05∗	0.19
	Y×S	16	4.27	<0.001∗∗∗	0.53

a *PEta.sq*: Partial Eta squared which used to measure the effect size of different variables in ANOVA models.

**Table 2:** One-way ANOVA of nutrients (N, P) and defensive compounds (tannin and total phenol) affected by decay year for each species (i.e., CWD type)

Species^a^	Components	*F*	*p value*	*Eta*^b^	*PEta.sq*^c^
PM	N	9.61	0.001∗∗∗	0.87	0.76
	P	17.30	<0.001∗∗∗	0.92	0.85
	Tannin	3.53	0.04∗	0.74	0.54
	Phenol	2.55	0.09	0.68	0.46
CL	N	37.31	<0.001∗∗∗	0.96	0.93
	P	33.11	<0.001∗∗∗	0.96	0.92
	Tannin	12.49	<0.001∗∗∗	0.90	0.81
	Phenol	16.81	<0.001∗∗∗	0.92	0.85
PE	N	5.78	<0.01∗∗	0.81	0.66
	P	72.53	<0.001∗∗∗	0.98	0.96
	Tannin	173.06	<0.001∗∗∗	0.99	0.98
	Phenol	5.85	<0.01∗∗	0.81	0.66
QG	N	8.03	<0.01∗∗	0.85	0.73
	P	100.17	<0.001∗∗∗	0.99	0.97
	Tannin	1.89	0.181	0.62	0.38
	Phenol	0.66	0.63	0.42	0.18
SS	N	24.24	<0.001∗∗∗	0.94	0.89
	P	103.46	<0.001∗∗∗	0.99	0.97
	Tannin	36.18	<0.001∗∗∗	0.96	0.92
	Phenol	17.93	<0.001∗∗∗	0.93	0.86

a PM: *Pinus massoniana*; CL: *Cunninghamia lanceolata*; PE: *Phyllostachys edulis*; QG: *Quercus glauca*; SS: *Schima superba*.

b Eta is the arithmetic square root of the Eta square.

c *PEta.sq*: Partial Eta squared which used to measure the effect size of different variables in ANOVA models.

As shown in Table 2, decay time (years since initial sampling) significantly influenced N and P concentrations (*p* < 0.01, *PEta.sq* = 0.66–0.97), explaining 66–97% of their variance. The dynamics of defensive compounds during decomposition were species-specific. Tannin and total phenol contents changed significantly over time in *Pinus massoniana* (tannin only), *Cunninghamia lanceolata*, *Phyllostachys edulis,* and *Schima superba* (*p* < 0.05, *PEta.sq* = 0.46–0.98), whereas no such trends were observed in *Quercus glauca*.

### Tannin and total phenol change dynamics

During decomposition, all species except *Quercus glauca* exhibited significant declines in tannins (−37.53% to −88.25%) and total phenolics (−37 to −85%) in the first year. *Quercus glauca* had the lowest initial concentrations of both compounds, which remained stable throughout decay. Tannin and phenolic compounds in deadwood were released during the first year for *Pinus massoniana*, *Phyllostachys edulis,* and *Schima superba*, and continuously decreased until the fifth year for *Cunninghamia lanceolata* (Figures 2 and 3). *Schima superba* displayed the highest initial levels of tannins and phenolics, coupled with the steepest first-year decline.

### Initial stem wood traits in relation to decay rate among different species

**Figure 4:**
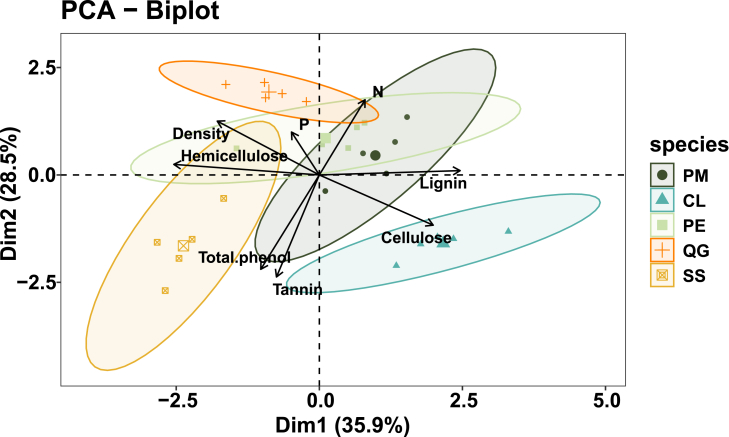
PCA of different tree species (i.e., CWD types) and influence factors including the wood density, C, N, P, cellulose, hemicellulose, lignin, tannin, and total phenol

Principal component analysis showed that the cumulative contribution of the first and second principal axes to the decay rate was 35.9% and 28.5%, respectively (Figure 4). The relatively fast decomposition species clustered in the left upper panel, showing that wood with high wood density, high P, and high hemicellulose characteristics is more decomposed.

### Nutrients and defensive components in relation to decay rates

**Figure 5:**
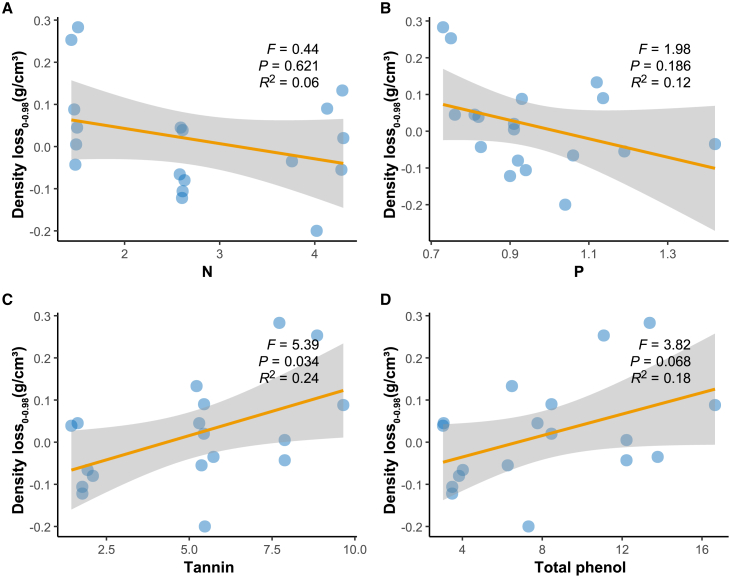
Analysis of the relationship between initial N, P, tannins, and total phenols and density loss from 0 to 0.98 years using linear mixed modeling (A) Linear mixed model between initial N and 0-0.98 year density loss. (B) Linear mixed model between initial P and 0-0.98 year density loss. (C) Linear mixed model between initial tannin and 0-0.98 year density loss. (D) Linear mixed model between initial total phenol and 0-0.98 year density loss.

**Figure 6:**
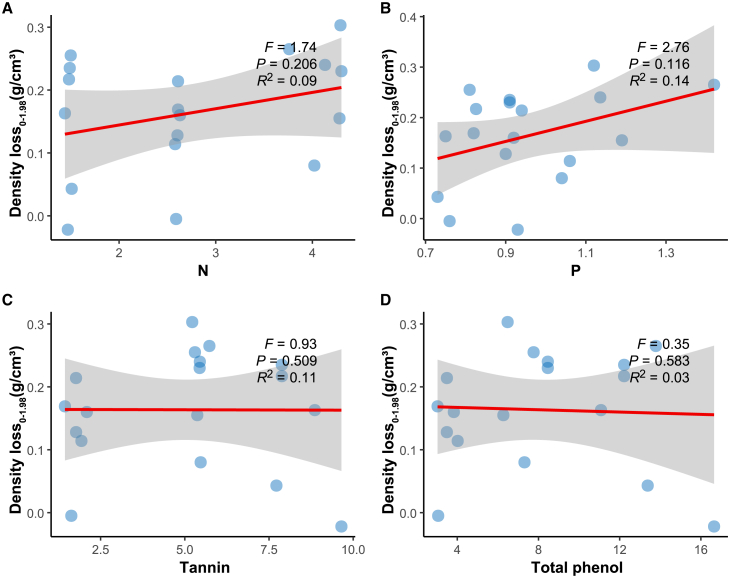
Analysis of the relationship between initial N, P, tannins, and total phenols and density loss from 0 to 1.98 years using linear mixed modeling (A) Linear mixed model between initial N and 0-1.98 year density loss. (B) Linear mixed model between initial P and 0-1.98 year density loss. (C) Linear mixed model between initial tannin and 0-1.98 year density loss. (D) Linear mixed model between initial total phenol and 0-1.98 year density loss.

**Figure 7:**
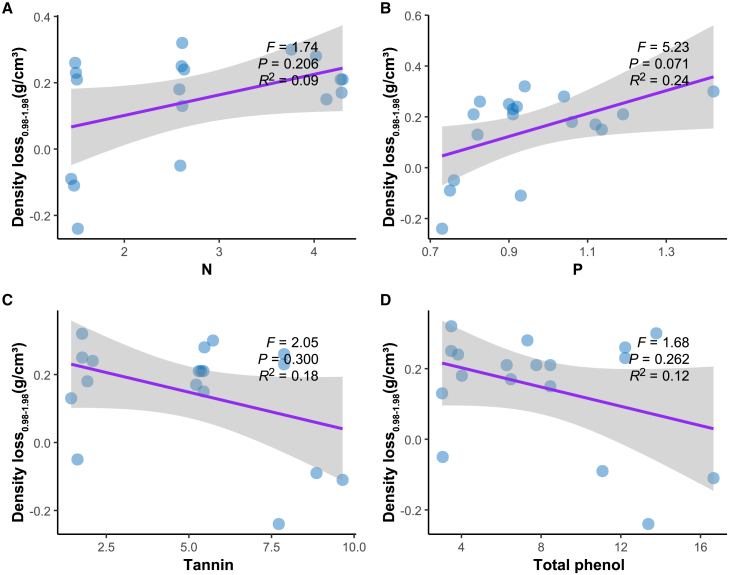
Analysis of the relationship between initial N, P, tannins and total phenols and density loss from 0.98 to 1.98 years using linear mixed modeling (A) Linear mixed model between initial N and 0.98-1.98 year density loss. (B) Linear mixed model between initial P and 0.98-1.98 year density loss. (C) Linear mixed model between initial tannin and 0.98-1.98 year density loss. (D) Linear mixed model between initial total phenol and 0.98-1.98 year density loss.

Over the 0–0.98 year period, mass loss rate was significantly and positively correlated only with initial tannin content in the three fast-decaying species (*Phyllostachys edulis, Quercus glauca, and Schima superba*) (Figure 5). The initial tannin content explained 24% of the density loss. However, N, P, and total phenolics showed no significant linear correlations during the 0–0.98 years (Figure 5), 0–1.98 years (Figure 6), or 0.98–1.98 years (Figure 7). Although not statistically significant, total phenolics trended similarly to tannins.

## Discussion

This study revealed significant interspecific variation in the CWD decomposition rates (k-value). Specifically, the broadleaved species *Quercus glauca* (k = 0.224 years^−1^) and *Schima superba* (k = 0.235 years^−1^) decomposed 1.4-fold faster than *Phyllostachys edulis* (k = 0.168 years^−1^). Coniferous species showed minimal mass loss over the five-year observation period, supporting our first hypothesis that angiosperm-derived CWD decays faster than conifer-derived CWD. A global synthesis of 295 k-value estimates revealed up to 244-fold variation in CWD decomposition rates, with species-level variation reaching 76-fold at individual sites. This aligns with previous findings that conifers exhibit significantly lower k-values than angiosperms.^2^ For bamboo, our results align with tropical forest studies showing moso bamboo decomposes more slowly than angiosperm trees.^35^

Previous studies have showed that initial N content in CWD is closely linked to interspecific decomposition rates variations.^36^ Higher initial N in plant residues stimulates microbial metabolism, growth, and reproduction, thereby accelerating substrate decomposition rates.^37^ However, *Schima superba* CWD decomposed fastest (Figure 3), indicating that factors other than initial N availability primarily regulate its decay. This rapid decomposition may be attributed to elevated microbial respiration, which converts CWD carbon into CO_2_, driving continuous decay. Additionally, N content increased significantly in all CWD species during decomposition, likely due to microbial N immobilization. In addition to N, P serves as a critical co-limiting nutrient in CWD decomposition.^36^ Our study found significant interspecific variation in the initial P content among CWD types. For instance, *Phyllostachys edulis* CWD (1.14 mg·g^−1^) had 61% higher P than *Cunninghamia lanceolata* (0.71 mg·g^−1^). This P availability difference correlated with decomposition rates, as higher P enhances microbial enzyme activity and substrate mineralization, accelerating mass loss. Among coniferous species, *Cunninghamia lanceolata* had lower P content and slower decay than *Pinus massoniana* (Figure 3), suggesting that available P may limit early decay in conifers. During decomposition, P followed a “release pattern,” likely due to nutrient release in the early stages of CWD decomposition.^37^

CWD consists of labile organic compounds (sugars, starches, proteins) and complex structural polymers (cellulose, hemicellulose, lignin). Interspecific variations in these components significantly influence CWD decomposition rates. During early decay, species-specific chemical profiles emerge due to CWD structural heterogeneity. As decomposition progresses and labile components are consumed, lignin becomes dominant, with the residual lignin concentration determining interspecific decay variation in later stages.^38^ Incorporating initial lignin content into decomposition models can greatly improve the accuracy of predicting decomposition rates (R^2^ = 0.55),^39^ consistent with this study’s findings. Cellulose is hydrolyzed by extracellular enzymes into small carbon compounds during pre-decomposition stages, which are easily absorbed and utilized by microorganisms.^40^ In contrast to other components, lignin is more difficult to decompose and therefore remains at a higher level in later stages of decomposition. This leads to an increase in the lignin/cellulose ratio over time.^41^ In this study, *Pinus massoniana*, *Cunninghamia lanceolata,* and *Phyllostachys edulis* with high initial lignin content exhibited lower decomposition rates compared to two angiosperms. Studies in the Indian Himalayan region showed litter C:N ratio and lignin content regulated the k-value,^42^ while in Karnataka’s southern dry agroclimatic zone, lignin content and the lignin:N ratio determined *Ficus benghalensis* litter decay rates.^43^ Lignin has a large and complex structure that makes it difficult to decompose. It may hinder the degradation of cellulose and hemicellulose embedded in cell wall structures containing lignins.^39^ On the other hand, most wood-degrading microorganisms primarily decompose cellulose; therefore, high levels of lignin prevent access for cellulose because wood-cell walls act as a physical barrier against non-lignocellulosic degrading microbes.^25^

**Figure 8:**
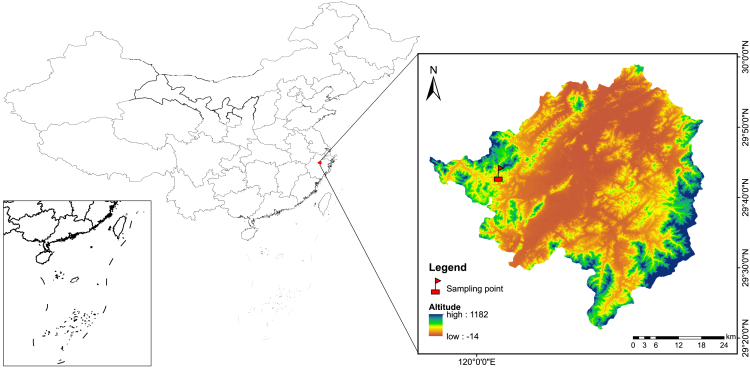
Geographical location of the study area

Phenols and tannins, as secondary metabolites, represent another type of recalcitrant compound besides lignin, widely present in CWD.^44^^,^^45^ In this study, tannin and phenol content in CWD species (except *Quercus glauca*, which maintained a low level) showed an overall decrease. Significant interspecific differences in initial tannin and phenol content were observed, consistent with previous studies on subtropical tree species.^46^ Contrary to our hypothesis, tannins and phenols did not persist in CWD. Gymnosperms had low initial levels, while the angiosperm *Schima superba,* with the highest tannin and phenol content, decomposed fastest. This may be related to the rapid decline in tannin and phenol content within 0.98 years in *Schima superba*. Except for *Quercus glauca*, all tree species showed a rapid decrease in these compounds by 0.98 years, likely due to early-stage leaching.^47^ The rainfall at the research site (Figure 8) is mainly concentrated from May to August, which enhances the leaching of phenolic compounds,^48^^,^^49^ promoting their degradation. Additionally, angiosperms attract more early-colonzing bacteria and fungi,^26^ including microbes such as *Staphylococcus, Klebsiella*, *Bacillus* that utilize tannins as a carbon source for growth,^50^^,^^51^ indicating context-dependent roles of tannin in decomposition. *Quercus glauca* showed no significant change in tannin and total phenolic content over five years, with a decomposition rate secod only to *Schima superba*, suggesting that it xylem anatomical and physical structure may override chemical effects on decay.

### Conclusions

In a five-year controlled experiment simulating tree dumping, we measured the decay rates of CWD from five dominant subtropical tree species. Results showed significant interspecific variation in decay rates. Two broadleaf species decomposed 40% and 18% faster than bamboo, respectively, while coniferous species decay more slowly. P availability limited decomposition in this subtropical region, whereas defensive tannins and phenolics had weak inhibitory effects during early decay. Our findings enhance the understanding of CWD-driven carbon and nutrient fluxes in subtropical forests. Species-specific k-value and nutrient release quantification improve the estimates of CWD contributions to long-term carbon sequestration and soil organic matter formation. In Chinese subtropical forests dominated by broadleaved trees, targeted management strategies can optimize deadwood carbon sequestration, especially during the early post-mortality phase after natural disturbances or large-scale tree dumping. As a crucial link between vegetation and soil carbon pools, CWD dynamics regulate forest carbon balance. Future research should elucidate the interactions among CWD, vegetation, and soil carbon pools to better quantify CWD’s role in the forest carbon cycle.

### Limitations of the study

Although this study conducted a 5-year *in situ* decomposition experiment to investigate interspecific CWD decay dynamics, integrating chemical analyses of cellulose, lignin, tannins, phenolics, and measurements of N and P nutrients, several limitations remain.

**First**, decay rates were calculated based on temporal changes in wood density, a method that overlooks volume loss from log morphological alterations during decomposition, potentially underestimating decay rates.

**Second**, bark, a common woody substrate covering tree trunks, exhibits distinct differences in nutrient concentration, density, structure, and function compared to wood. However, in experimental designs, bark and wood are often treated as a homogeneous substrate. This simplification hinders our understanding of how bark properties affect the decomposition processes of both bark and wood.

## Resource availability

### Lead contact

Further information and requests for resources and reagents should be directed to and will be fulfilled by the lead contact, Nan Wang (wangnan198110@163.com).

### Materials availability

This study did not generate new unique reagents.

### Data and code availability

• Data: The data reported in this article are available within the main text or supplemental information and will be shared by the lead contact upon request.
• Code: This article does not report original code.
• Other items: Any additional information required to reanalyze the data reported in this article will be shared by the lead contact upon request.

## Acknowledgments

Research Foundation of Shaoxing 330 Overseas Talents Program.

## Author contributions

N. W. and X. W. designed the study. T. X. performed the data analysis and statistical analyses. T. X. and H. C. performed experiments. S. B. assisted in carrying out the experiments. T. X. and N. W. wrote the article.

## Declaration of interests

The authors declare no competing interests.

## STAR★Methods

### Key resources table

**Table:** 

REAGENT or RESOURCE	SOURCE	IDENTIFIER
**Chemicals, peptides, and recombinant proteins**		

K_2_SO_4_	Energy Chemical Ltd.	Cat# 7778-80-5
NaOH	Energy Chemical Ltd.	Cat# 1310-73-2
Na_2_CO_3_	Energy Chemical Ltd.	Cat# 497-19-8
H_3_P(Mo_3_O_10_)_4_·xH_2_O	Energy Chemical Ltd.	Cat# 51429-74-4
Na_2_WO_4_·2H_2_O	Energy Chemical Ltd.	Cat# 10213-10-2
CuSO4·5H_2_O	Energy Chemical Ltd.	Cat# 7757-99-8
DNS	Energy Chemical Ltd.	Cat# 34490-86-3

**Software and algorithms**		

R version 4.3.1	R Core Team	https://www.r-project.org/

### Method details

#### Study area and site

The study was conducted in Wuxie National Forest Park (29°72′N, 120°05′E) in Zhuji City, Zhejiang Province, China, with average elevation of 220 m (Figure 8). This area belongs to the subtropical monsoon climate zone, with warm and rainy summer and cold winter. The average annual air temperature is 16.3°C, the average annual rainfall is 1374 mm. The average annual rainfall is 158.3 days, mainly concentrated from May to August (70% occurred in this period). The soil type is mountain red and yellow, with pH of 4.5-5.3. The park’s land-use types include cropland, orchards, grassland, and forestland. The forestland spans 534 hectares, mainly comprising subtropical evergreen broadleaf forest, mixed conifer-broadleaf forest, and Moso bamboo forest. Evergreen broadleaf and mixed conifer-broadleaf forests together cover 377 hectares, accounting for 74.7% of the total forest area.

#### Experimental design and sample collection

In January 2018, mature trees of five species (*Pinus massoniana*, *Cunninghamia lanceolata*, *Quercus glauca*, *Schima superba* and *Phyllostachys edulis*), each in 8-10 individuals were harvested from the forest at the study site. The trees are naturally growing and healthy. The trunks were then cut into 1m-long logs and laid flat on the ground (as down wood). In this study, CWD was categorized by its origin species, with five CWD types representing the dominant coniferous and broadleaved trees in the study area. To maintain consistency and clarity, “CWD type” and “tree species” are used interchangeably in the text, tables, and figures. For example, the notation "tree species (i.e., CWD type)" is utilized to highlight their equivalence. Samples were then taken from the felled logs at yearly intervals at 0, 0.98, 2.39, 3.23 and 4.81 years. At the time of collection, 5-10 cm wood disk were cut from the logs and taken to the laboratory in self-sealing bags. The samples were dried naturally for five days, then nearly 1/4 of each sample was taken to measure dry weight and volume (drainage method). The bulk density can be defined as:

(Equation 1) $$\rho =\frac{M}{V}$$

where, M is the dry weight of the sample (g); V is the volume of the sample (cm^3^); and ρ is the bulk density of the sample (g/cm^3^).

Air-dried samples were oven-dried at 70°C until reaching a constant weight.^52^ The oven-dried materials were homogenized by ball-mill grinding to produce a fine powder for the quantitative analysis of chemical constituents.

#### Chemical analysis

The N content was analyzed by the semi-Kjeldahl method^53^ and P by atomic absorption spectrometry method.^54^ The total phenolic content was determined by the Folin–Ciocalteu method.^55^ Under alkaline conditions, the phenolic substances reduce tungstic molybdic acid to produce a blue compound with a characteristic absorption peak at 760 nm, and the absorbance value was read to calculate the total phenol content. The tannin content was determined by the microplate method.^56^^,^^57^ Tannins reduce phosphomolybdic acid under alkaline conditions to form a blue compound, and the resulting absorbance was measured at 650 nm. The lignocellulose fractions of the wood were also determined, these data are listed in the Appendix (Table S1; Figure S1), the data were only used for the final integrated analysis. Lignin was determined by the acetylation method.^58^^,^^59^ The cellulose content was determined by the anthrone colorimetric method.^60^ The xylose content was determined by colorimetric method,^61^ and then the hemicellulose content was calculated.

### Quantification and statistical analysis

The wood decay rates among different tree species were calculated by wood loss estimated by wood density (WD) change with decay years. The negative exponential decay models were fitted separately for different trees species to estimate how wood density change with decay years.

(Equation 2) $$Y=A*e^{-kt}$$

where, *t* is time in years; *A* and *k* are the estimate of initial density and decay rate constant (/year) of the model, respectively. Parameter estimates are not statistically significant when *P* value >0.05 or low R^2^.

Two-way ANOVA was used to analyze the effects of tree species, decomposition time and their interactions on N, P, tannin and total phenol contents in CWD. One-way ANOVA was used to analyze the effects of decomposition time on their changes in each tree species, and the value of the Eta-squared was calculate.^62^ Principal Component Analysis (PCA) was used to detect differences in initial wood properties between species. Raw variables were Z-score standardized (mean = 0, SD = 1). Subsequently, a covariance matrix was calculated from the standardized data and subjected to eigenvalue decomposition to obtain eigenvalues and eigenvectors. Based on the Kaiser-Harris criterion, principal components with eigenvalues greater than 1 were selected for further analysis. To improve interpretability, Varimax rotation was performed to maximize variance in loading matrices, ensuring that each principal component was predominantly characterized by a reduced number of variables. Linear mixed model (LMM) was conducted to analyze the relationship between density loss rate in the first two years (because fast-decay species lost large mass in first two years), in relation to N, P, tannin and total phenol at initial (year 0) and the later (year 0.98). The LMM is an extension of the multiple linear models where covariates are assumed to have a linear relationship to the dependent variable. It is normally used when data is not independent. This model is considered more realistic or logical compare to a traditional linear model which only considers the fixed effects. Data analysis was conducted using R software, version 4.2.1 with RStudio. Originlab 2021 was used for plotting.

## References

[1] Cao Y., You W.B., Wang F.Y., Wu L.Y., He D.J. (2021). Research progress on carbon storage of coarse woody debris in forest ecosystems. Acta Ecol. Sin..

[2] Harmon M.E., Fasth B.G., Yatskov M., Kastendick D., Rock J., Woodall C.W. (2020). Release of coarse woody detritus-related carbon: a synthesis across forest biomes. Carbon Bal. Manag..

[3] Herrmann S., Kahl T., Bauhus J. (2015). Decomposition dynamics of coarse woody debris of three important central European tree species. For. Ecosyst..

[4] Freschet G.T., Weedon J.T., Aerts R., van Hal J.R., Cornelissen J.H.C. (2012). Interspecific differences in wood decay rates: insights from a new short-term method to study long-term wood decomposition. J. Ecol..

[5] Chen J., Duan W.B., Qu M.X. (2021). Effects of uprooted treefalls and their microsites on decomposition rate and nutrient release of litters in Picea koraiensis-Abies nephrolepis-Pinus koraiensis forest. Acta Ecol. Sin..

[6] Bilous A., Matsala M., Radchenko V., Matiashuk R., Boyko S., Bilous S. (2019). Coarse woody debris in mature oak stands of Ukraine: carbon stock and decomposition features. IDEAS.

[7] Hu H., Wang S., Guo Z., Xu B., Fang J. (2015). The stage-classified matrix models project a significant increase in biomass carbon stocks in China’s forests between 2005 and 2050. Sci. Rep..

[8] Russell M.B., Fraver S., Aakala T., Gove J.H., Woodall C.W., D’Amato A.W., Ducey M.J. (2015). Quantifying carbon stores and decomposition in dead wood: A review. For. Ecol. Manage..

[9] Pan Y., Birdsey R.A., Fang J., Houghton R., Kauppi P.E., Kurz W.A., Phillips O.L., Shvidenko A., Lewis S.L., Canadell J.G. (2011). A Large and Persistent Carbon Sink in the World’s Forests. Science.

[10] Shorohova E., Kapitsa E., Kuznetsov A., Kuznetsova S., Lopes de Gerenyu V., Kaganov V., Kurganova I. (2022). Coarse woody debris density and carbon concentration by decay classes in mixed montane wet tropical forests. Biotropica.

[11] Stokland J.N., Siitonen J., Jonsson B.G. (2012).

[12] Wang Z., Xu M., Li F., Bai Y., Hou J., Li X., Cao R., Deng Y., Jiang Y., Wang H., Yang W. (2023). Changes in soil bacterial communities and functional groups beneath coarse woody debris across a subalpine forest successional series. Glob. Ecol. Conserv..

[13] Bani A., Pioli S., Ventura M., Panzacchi P., Borruso L., Tognetti R., Tonon G., Brusetti L. (2018). The role of microbial community in the decomposition of leaf litter and deadwood. Appl. Soil Ecol..

[14] Merganičová K., Merganič J. (2010). Coarse woody debris carbon stocks in natural spruce forests of Babia hora. J. For. Sci. (Prague)..

[15] Carmona M.R., Armesto J.J., Aravena J.C., Pérez C.A. (2002). Coarse woody debris biomass in successional and primary temperate forests in Chiloé Island, Chile. For. Ecol. Manage..

[16] Yang F.F., Li Y.L., Zhou G.Y., Wenigmann K.O., Zhang D.Q., Wenigmann M., Liu S.Z., Zhang Q.M. (2010). Dynamics of coarse woody debris and decomposition rates in an old-growth forest in lower tropical China. For. Ecol. Manage..

[17] Iwashita D.K., Litton C.M., Giardina C.P. (2013). Coarse woody debris carbon storage across a mean annual temperature gradient in tropical montane wet forest. For. Ecol. Manage..

[18] Bradford M.A., Veen G.F.C., Bradford E.M., Covey K.R., Crowther T.W., Fields N., Frankson P.T., González-Rivero J., Jevon F.V., Kuebbing S.E. (2023). Coarse woody debris accelerates the decomposition of deadwood inputs across temperate forest. Biogeochemistry.

[19] Dale V.H., Joyce L.A., McNulty S., Neilson R.P., Ayres M.P., Flannigan M.D., Hanson P.J., Irland L.C., Lugo A.E., Peterson C.J. (2001). Climate Change and Forest Disturbances: Climate change can affect forests by altering the frequency, intensity, duration, and timing of fire, drought, introduced species, insect and pathogen outbreaks, hurricanes, windstorms, ice storms, or landslides. Bioscience.

[20] Silva L.F.S.G., de Castilho C.V., de Oliveira Cavalcante C., Pimentel T.P., Fearnside P.M., Barbosa R.I. (2016). Production and stock of coarse woody debris across a hydro-edaphic gradient of oligotrophic forests in the northern Brazilian Amazon. For. Ecol. Manage..

[21] Chambers J.Q., Negron-Juarez R.I., Marra D.M., Di Vittorio A., Tews J., Roberts D., Ribeiro G.H.P.M., Trumbore S.E., Higuchi N. (2013). The steady-state mosaic of disturbance and succession across an old-growth Central Amazon forest landscape. Proc. Natl. Acad. Sci. USA.

[22] Chen H., Xu Z.B. (1991). History, Current Situation and Tendency of CWD Ecological Research. Chinese J. Ecol..

[23] Viitanen H., Toratti T., Makkonen L., Peuhkuri R., Ojanen T., Ruokolainen L., Räisänen J. (2010). Towards modelling of decay risk of wooden materials. Eur. J. Wood Prod..

[24] Cornwell W.K., Cornelissen J.H.C., Amatangelo K., Dorrepaal E., Eviner V.T., Godoy O., Hobbie S.E., Hoorens B., Kurokawa H., Pérez-Harguindeguy N. (2008). Plant species traits are the predominant control on litter decomposition rates within biomes worldwide. Ecol. Lett..

[25] Cornwell W.K., Cornelissen J.H.C., Allison S.D., Bauhus J., Eggleton P., Preston C.M., Scarff F., Weedon J.T., Wirth C., Zanne A.E. (2009). Plant traits and wood fates across the globe: rotted, burned, or consumed?. Glob. Change Biol..

[26] Weedon J.T., Cornwell W.K., Cornelissen J.H.C., Zanne A.E., Wirth C., Coomes D.A. (2009). Global meta-analysis of wood decomposition rates: a role for trait variation among tree species?. Ecol. Lett..

[27] Dix N.J., Webster J. (1995).

[28] Pearce R.B. (1996). Antimicrobial defences in the wood of living trees. New Phytol..

[29] Cortez J., Garnier E., Pérez-Harguindeguy N., Debussche M., Gillon D. (2007). Plant traits, litter quality and decomposition in a Mediterranean old-field succession. Plant Soil.

[30] Cheng C., Liu Z., Zhang Y., He Q., Li B., Wu J. (2024). Leaf litter decomposition and its drivers differ between an invasive and a native plant: Management implications. Ecol. Appl..

[31] Ganjegunte G.K., Condron L.M., Clinton P.W., Davis M.R., Mahieu N. (2004). Decomposition and nutrient release from radiata pine (Pinus radiata) coarse woody debris. For. Ecol. Manage..

[32] Zhou H.C., Kang H.X., Wei J., Gao C.J., Hussain M., Fu Y.J., Li M.D., Li F.L., Xu S.J.L., Lee F.W.F. (2023). Microcosm study on fate and dynamics of mangrove tannins during leaf litter leaching. Ecol. Process..

[33] Krishna M.P., Mohan M. (2017). Litter decomposition in forest ecosystems: a review. Energy Ecol. Environ..

[34] Liese W. (1998). The anatomy of bamboo culms. International Network for Bamboo and Rattan (INBAR).

[35] Liu G., Cornwell W.K., Cao K., Hu Y., Van Logtestijn R.S.P., Yang S., Xie X., Zhang Y., Ye D., Pan X. (2015). Termites amplify the effects of wood traits on decomposition rates among multiple bamboo and dicot woody species. J. Ecol..

[36] Chen Y., Sayer E.J., Li Z., Mo Q., Li Y., Ding Y., Wang J., Lu X., Tang J., Wang F. (2016). Nutrient limitation of woody debris decomposition in a tropical forest: contrasting effects of N and P addition. Funct. Ecol..

[37] Harmon M.E., Franklin J.F., Swanson F.J., Sollins P., Gregory S.V., Lattin J.D., Anderson N.H., Cline S.P., Aumen N.G., Sedell J.R. (1986). Ecology of Coarse Woody Debris in Temperate Ecosystems. Adv. Ecol. Res..

[38] Strukelj M., Brais S., Quideau S.A., Angers V.A., Kebli H., Drapeau P., Oh S.W. (2013). Chemical transformations in downed logs and snags of mixed boreal species during decomposition. Can. J. For. Res..

[39] Van Geffen K.G., Poorter L., Sass-Klaassen U., van Logtestijn R.S.P., Cornelissen J.H.C. (2010). The trait contribution to wood decomposition rates of 15 Neotropical tree species. Ecology.

[40] Wang Y., Liu Q., Yan L., Gao Y., Wang Y., Wang W. (2013). A novel lignin degradation bacterial consortium for efficient pulping. Bioresour. Technol..

[41] Crawford R.L. (1981).

[42] Ahirwal J., Saha P., Nath A., Nath A.J., Deb S., Sahoo U.K. (2021). Forests litter dynamics and environmental patterns in the Indian Himalayan region. For. Ecol. Manage..

[43] Dhanya B., Viswanath S., Purushothaman S. (2013). Decomposition and Nutrient Release Dynamics of Ficus benghalensis L. Litter in Traditional Agroforestry Systems of Karnataka, Southern India. ISRN Forestry.

[44] Preston C.M., Nault J.R., Trofymow J.A., Smyth C. (2009). Chemical Changes During 6 Years of Decomposition of 11 Litters in Some Canadian Forest Sites. Part 1. Elemental Composition, Tannins, Phenolics, and Proximate Fractions. Ecosystems.

[45] Stutz K.P., Dann D., Wambsganss J., Scherer-Lorenzen M., Lang F. (2017). Phenolic matter from deadwood can impact forest soil properties. Geoderma.

[46] Ristok C., Leppert K.N., Franke K., Scherer-Lorenzen M., Niklaus P.A., Wessjohann L.A., Bruelheide H. (2017). Leaf litter diversity positively affects the decomposition of plant polyphenols. Plant Soil.

[47] Du T., Chen Y.L., Bi J.H., Yang Y.T., Zhang L., You C.M., Tan B., Xu Z.F., Wang L.X., Liu S.N., Li H. (2023). Effects of forest gap on losses of total phenols and condensed tannins of foliar litter in a subalpine forest of western Sichuan, China. Chinese J. Plant Ecol..

[48] Brandt L.A., King J.Y., Hobbie S.E., Milchunas D.G., Sinsabaugh R.L. (2010). The Role of Photodegradation in Surface Litter Decomposition Across a Grassland Ecosystem Precipitation Gradient. Ecosystems.

[49] Shure D.J., Mooreside P.D., Ogle S.M. (1998). Rainfall effcets on Plant–Eherbivore processes in anupland oak forest. Ecology.

[50] Ascacio-Valdés J.A., Buenrostro J.J., De la Cruz R., Sepúlveda L., Aguilera A.F., Prado A., Contreras J.C., Rodríguez R., Aguilar C.N. (2014). Fungal biodegradation of pomegranate ellagitannins. J. Basic Microbiol..

[51] Alshammari N., Fraih A.B.Z., Al Tami M.S., Upadhyay T.K. (2023). Wood-decay fungi associated with decaying of coarse woody debris in Hail region, North of Saudi Arabia. Cell. Mol. Biol..

[52] Schofield J.A., Hagerman A.E., Harold A. (1998). Loss of tannins and other phenolics from willow leaf litter. J. Chem. Ecol..

[53] Uselman S.M., Qualls R.G., Lilienfein J. (2012). Quality of soluble organic C, N, and P produced by different types and species of litter: Root litter versus leaf litter. Soil Biol. Biochem..

[54] Ashagrie Y., Zech W., Guggenberger G. (2005). Transformation of a Podocarpus falcatus dominated natural forest into a monoculture Eucalyptus globulus plantation at Munesa, Ethiopia: soil organic C, N and S dynamics in primary particle and aggregate-size fractions. Agric. Ecosyst. Environ..

[55] Maduwanthi S.D.T., Marapana R.A.U.J. (2021). Total phenolics, flavonoids and antioxidant activity following simulated gastro-intestinal digestion and dialysis of banana (Musa acuminata, AAB) as affected by induced ripening agents. Food Chem..

[56] Diouf P.N., Merlin A., Perrin D. (2006). Antioxidant properties of wood extracts and colour stability of woods. Ann. For. Sci..

[57] Huang Z., Hashida K., Makino R., Kawamura F., Shimizu K., Kondo R., Ohara S. (2009). Evaluation of biological activities of extracts from 22 African tropical wood species. J. Wood Sci..

[58] Chang X.F., Chandra R., Berleth T., Beatson R.P. (2008). Rapid, Microscale, Acetyl Bromide-Based Method for High-Throughput Determination of Lignin Content in Arabidopsis thaliana. J. Agric. Food Chem..

[59] Fukushima R.S., Kerley M.S., Ramos M.H., Porter J.H., Kallenbach R.L. (2015). Comparison of acetyl bromide lignin with acid detergent lignin and Klason lignin and correlation with in vitro forage degradability. Anim. Feed Sci. Technol..

[60] Viles F.J., Silverman L. (1949). Determination of Starch and Cellulose with Anthrone. Anal. Chem..

[61] Kuyper M., Toirkens M.J., Diderich J.A., Winkler A.A., van Dijken J.P., Pronk J.T. (2005). Evolutionary engineering of mixed-sugar utilization by a xylose-fermenting strain. FEMS Yeast Res..

[62] Norouzian R., Plonsky L. (2018). Eta-and partial eta-squared in L2 research: A cautionary review and guide to more appropriate usage. Second Lang. Res..

