# Supplementary material for: Intrinsic nutrients and defensive compounds drive coarse woody debris decay in five dominant subtropical tree species, China
Source: iScience. 2025 Jun 19;28(7):112961. doi: 10.1016/j.isci.2025.112961 (PMC12270689; doi:10.1016/j.isci.2025.112961)
Supplement: Document S1. Figure S1 and Table S1 [file mmc1.pdf]

iScience, Volume *28*

Supplemental information

Intrinsic nutrients and defensive compounds drive coarse woody debris decay in five dominant subtropical tree species, China

Xiaoyu Wang, Tingsi Xie, Hui Chen, Shangbin Bai, and Nan Wang

Table S1. The initial density, C, N, P, cellulose, hemicellulose, lignin, tannin and total phenol content of (mean ± SD) in different tree species (i.e., CWD types)

Traits	PM	CL	PE	QG	SS	*P* value
Density (g·cm-3)	0.43±0.07b	0.31±0.04c	0.62±0.13a	0.57±0.05)a	0.58±0.10a	<0.001***
C (%)	48.74±1.31ab	50.38±1.17a	40.41±0.72b	45.14±0.83ab	43.07±0.11ab	0.0182 *
N (mg/g)	3.07±0.33b	2.06±0.28d	4.13±0.24a	2.60±0.02c	1.48±0.03e	<0.001***
P (mg/g)	0.91±0.31ab	0.71±0.17b	1.14±0.19a	0.9±0.12ab	0.83±0.09ab	0.0281 *
Hemicellulose (mg/g)	227.86±9.9b	207.55±30.51b	237.62±43.19b	314.71±21.63a	324.86±11.01a	<0.001***
Cellulose (mg/g)	483.44±4.64ab	528.46±5.25a	497.97±5.36ab	435.66±5.50c	467.12±2.84bc	<0.001***
Lignin (mg/g)	265.00±25.46b	307.59±14.8a	245.41±15.00b	236.49±18.44b	179.49±16.83c	<0.001***
Tannin(mg/g)	4.26±0.93c	6.12±0.73b	5.45±0.19b	1.77±0.25d	8.28±0.95a	<0.001***
Total phenol(mg/g)	6.56±2.55b	8.54±1.04b	7.46±1.13b	3.49±0.45c	12.22±1.31a	<0.001***

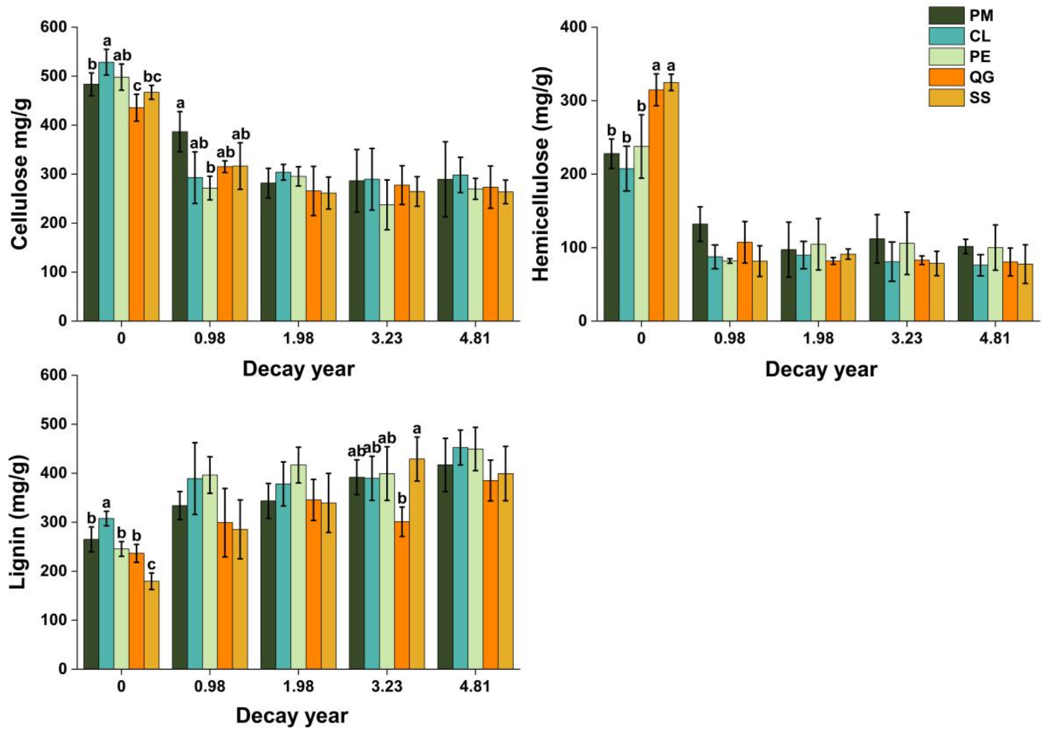

Figure S1. The cellulose, hemicellulose and lignin content (mean ± SD) in different tree species (i.e., CWD types) along with decay progress. Different lower letters indicate statistically significant differences ($P < 0.05$) in the indicators among different tree species
